# Organizational readiness for change and work process of oral health teams in Primary Health Care: an exploratory study in the initial implementation phase of MonitoraSB, Minas Gerais, Brazil, 2024

**DOI:** 10.1590/S2237-96222025v34e20240802.en

**Published:** 2025-10-20

**Authors:** Maria Edileusa Santos, Raquel Conceição Ferreira, Elisa Lopes Pinheiro, Samira Suelen Andrade Vieira, Maria Inês Barreiros Senna

**Affiliations:** 1 Universidade Federal de Minas Gerais, Belo Horizonte, MG, Brazil; 2 Universidade Federal de Minas Gerais, Departamento de Odontologia Social e Preventiva, Belo Horizonte, MG, Brazil; 3 Universidade Federal de Minas Gerais, Departamento de Clínica, Patologia e Cirurgia Odontológicas, Belo Horizonte, MG, Brazil

**Keywords:** Health Services Research, Dental Health Services, Dental Care Team, Process Assessment, Health Care, Evaluation Study, Investigación sobre Servicios de Salud, Servicios Salud Dental, Equipo de Atención Dental, Evaluación de Procesos, Atención de Salud, Estudio de Evaluación

## Abstract

**Objective:**

To investigate association between individual characteristics and organizational readiness for change and the work process of oral health teams.

**Methods:**

This was an exploratory study conducted during the alignment phase of the implementation of MonitoraSB, a proposal for monitoring oral health services in Primary Health Care, conducted in 13 cities in Minas Gerais state, which covered 86 oral health teams. The work process of these teams was evaluated as an outcome related to health services through a self-assessment questionnaire. Determinants of implementation (individual characteristics and organizational readiness for change) were assessed based on the Consolidated Framework for Implementation Research. Relative frequencies were obtained for categorical variables and median and interquartile range for readiness scores. Latent class analysis grouped the teams according to the work process. Associations were investigated using the chi-square test.

**Results:**

A total of 74 teams with 163 professionals participated. The teams were classified as having “elementary work processes” (49.6%) or “consolidated work processes” (50.4%) and presented high organizational readiness scores (median: 51; interquartile range: 11). The highest percentage of teams with consolidated work processes was found in those comprised of professionals with temporary contracts (66.1%), compared to teams with permanent employees (44.7%) (p-value 0.011), and in those having professionals who presented high effectiveness (60.8%) compared to those with low effectiveness (44.7%) (p-value 0.049).

**Conclusion:**

Teams with greater organizational readiness and less stable employment relationships presented more consolidated work processes more frequently. Organizational readiness should be taken into consideration with the aim of favoring implementation of monitoring initiatives, such as MonitoraSB.

## Resumen

**Objetivo:**

Investigar la asociación entre las características individuales y la preparación organizacional para el cambio con el proceso de trabajo de los equipos de salud bucal.

**Métodos:**

Estudio exploratorio realizado durante la fase de alineación de la implementación de MonitoraSB, una propuesta para el monitoreo de los servicios de salud bucal en la Atención Primaria de Salud, realizada en 13 ciudades de Minas Gerais, que abarcó 86 equipos de salud bucal. El proceso de trabajo de estos equipos se evaluó como un resultado relacionado con los servicios de salud mediante un cuestionario de autoevaluación. Los determinantes de la implementación (características individuales y preparación organizacional para el cambio) se evaluaron con base en el Consolidated Framework for Implementation Research. Se obtuvieron frecuencias relativas para las variables categóricas y la mediana y el rango intercuartil para las puntuaciones de preparación. El análisis de clases latentes agrupó a los equipos según el proceso de trabajo. Las asociaciones se investigaron mediante la prueba de chi-cuadrado.

**Resultados:**

Participaron 74 equipos con 163 profesionales. Los equipos se clasificaron como con “procesos de trabajo elementales” (49,6%) y “consolidados” (50,4%) y presentaron puntuaciones altas de preparación organizacional (mediana: 51; rango intercuartil: 11). El mayor porcentaje de equipos con procesos de trabajo consolidados se observó en aquellos cuyos profesionales fueron contratados (66,1%), en comparación con los equipos con empleados permanentes (44,7%) (p-valor 0,011), y en aquellos cuyos profesionales presentaron alta efectividad (60,8%) en comparación con aquellos con baja efectividad (44,7%) (p-valor 0,049).

**Conclusión:**

Los equipos con mayor preparación organizacional y relaciones laborales menos estables presentaron con mayor frecuencia procesos de trabajo más consolidados. La preparación organizacional debe considerarse para favorecer la implementación de iniciativas de monitoreo, como MonitoraSB.

**Ethical aspects**

This research respected ethical principles, having obtained the following approval data:

Research Ethics Committee: Universidade Federal de Minas Gerais

Opinion number: 5,326,307

Approval date: 1/4/2022

Certificate of Submission for Ethical Appraisal: 55573922.3.0000.5149

Informed Consent Form: All participants signed the informed consent record.

## Introduction

Provision and funding of oral health care are globally affected by lack of indicators for monitoring and evaluating services (1). In Brazil, these indicators are limited and have been impacted by changes in Primary Health Care evaluation initiatives over time (2). MonitoraSB emerges as an innovation for monitoring and evaluating oral health services in Primary Health Care that seeks to fill this gap (2). It consists of a matrix of 54 indicators prepared from data made available by the Primary Care Health Information System and by digital tools, namely the interactive panel and the calculator (3,4).

Monitoring and evaluation are guidelines of the National Oral Health Policy for improving the services provided by the Brazilian National Health System (5). Oral health teams are required to generate, input and ensure the quality of records held on health information systems. In addition, they are required to participate in meetings to monitor and discuss the planning and evaluation of team actions, using available data to adjust their work process (6).

In the process of implementing MonitoraSB the participation of health professionals as co-responsible and active subjects in the monitoring process is expected (3). The team analyzes and interprets the information provided by the MonitoraSB indicators and participates in the discussion and planning of actions to address the difficulties revealed by monitoring, with the aim of improving the care provided (3). This choice assumes that health work is characterized by a collective, collaborative and institutional process (5), guided by informed decision-making about the needs of the population and service performance (7).

In implementation research, health service outcomes can reveal the effect of adopting new practices or interventions (2). Thus, the work process of oral health teams can be an outcome sensitive to interventions aimed at monitoring indicators (8). The work process of these teams can vary according to the characteristics of the professionals, services and demands (5,8).

Identifying determinants of implementation aims to understand the barriers and facilitators for the effective implementation of innovation (9). The Consolidated Framework for Implementation Research is one of the most widely used frameworks for this purpose (9). It presents 37 constructs organized into five domains: characteristics of the intervention, outer setting, inner setting, characteristics of the individuals involved and implementation process (10).

The inner setting encompasses structural characteristics, network of relationships, communication, culture, climate, and organizational readiness, which may interact and impact the success of implementation (10). Organizational readiness for change is a multilevel construct that reflects the psychological and behavioral state shared by a team and is related to commitment and the ability to implement changes (11). In addition to being a predictive factor for the success of implementation, this readiness is dynamic and can be modified during the process (10-12). Despite its relevance, it is little studied among oral health professionals (11,13,14). Individual characteristics, such as knowledge and beliefs about the intervention, identification with the organization, and other personal attributes, also affect implementation (10). The determinants involved in implementation may be transitory and subject to change, which highlights the importance of monitoring them (10).

Characterizing the individual and contextual determinants of implementation and the work process of teams is essential in the initial phase of MonitoraSB implementation (2). This analysis can guide the development of effective strategies for its successful implementation, promoting changes in the work process (2).

The objective of this study was to describe the work process of oral health teams in the initial phase of MonitoraSB implementation and to characterize these professionals in terms of their demographic profile, professional performance, and organizational readiness for change. In addition, the association of individual characteristics and organizational readiness for change with the work process of teams was investigated. The hypothesis tested was that a higher percentage of teams with more consolidated work processes would be found when their professionals presented higher qualifications, stable employment relationships, and greater organizational readiness for change.

## Methods

### Study design and sample

**Figure 1:**
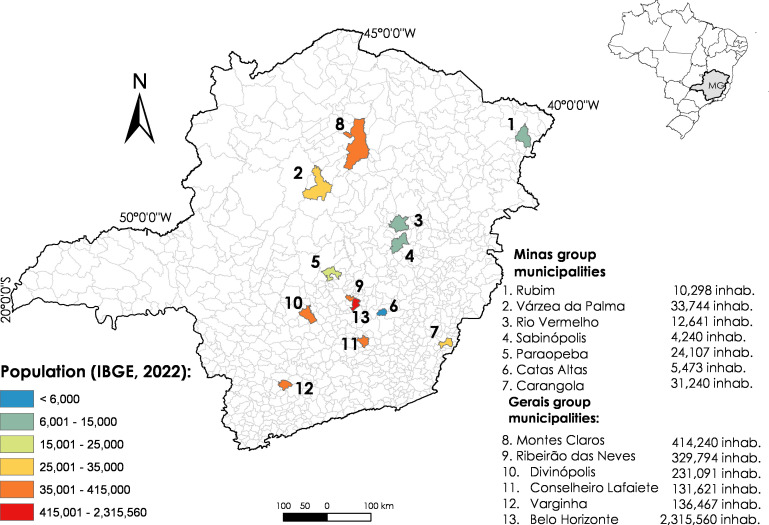
Municipalities of the Minas and Gerais groups participating in the MonitoraSB implementation survey. Minas Gerais, 2022

This was an exploratory study based on data from the initial phase of the MonitoraSB implementation research, approved by the Research Ethics Committee of the Universidade Federal de Minas Gerais. Mixed methods were used and the study is ongoing in 13 municipalities in Minas Gerais (12 inner state municipalities and the state capital). The municipalities were selected by convenience, considering partnerships with the School of Dentistry of the Universidade Federal de Minas Gerais and municipalities of origin of graduates of the professional master’s degree course in dentistry in public health. The municipalities were grouped by population size, according to 2022 data from the Brazilian Institute of Geography and Statistics, and classified into the Minas group (up to 40,000 inhabitants) and the Gerais group (population with more than 100,000 inhabitants) (Figure 1).

The sample was comprised of 86 oral health teams indicated by municipal health service managers and totaled 186 professionals, including dentists and oral health technicians and auxiliaries.

### Variables and data source 

The work process of oral health teams was the outcome related to health services, analyzed by a self-assessment instrument based on the guidelines of the National Oral Health Policy, the National Primary Care Policy, the Self-Assessment Booklet for Improving Access and Quality of Primary Care, and the Program for Primary Care Access and Quality Improvement (2). The instrument was validated by 19 experts, using the modified Delphi technique, with a content validity index >75% (3). The instrument predefined quality standards to assess the performance of the teams (15) and consisted of 37 statements organized into two dimensions and seven subdimensions, according to the model for assessing the effectiveness of oral health care adopted by MonitoraSB (3). Each standard was accompanied by a statement, and the team professional selected “yes” or “no”, according to the achievement of the standard. This instrument was answered by one of each team’s professionals.

The determinants of implementation (individual characteristics and organizational readiness for change) were assessed by all professionals on the teams. The individual characteristics analyzed were: sex, age, professional category (dental surgeon, oral health technician, oral health auxiliary), education (high school education, technical education, graduation, postgraduate studies), employment relationship (temporary contract, permanent contract) and length of service at the institution. Organizational readiness was assessed using the validated Brazilian version of the Organizational Readiness for Implementing Change questionnaire (3,16), consisting of 11 statements on a 5-point Likert scale (“1, I completely disagree” to “5, I completely agree”). The organizational readiness for change score was obtained by adding the total score (score between 11 and 55) and the questions related to the commitment construct (score between 5 and 25) and the effectiveness construct (score between 6 and 30). 

### Data collection and bias minimization procedures 

Data collection took place from March 2023 to February 2024. The instruments were sent individually by email to each professional and shared via WhatsApp by local focal points, these being health professionals who coordinate the implementation of MonitoraSB in the municipalities. Reminders were sent to increase participation and adherence of those involved. The questionnaires contained detailed instructions for correct completion. During collection, local focal points and the study’s researchers were available to clarify doubts.

### Statistical analysis 

The initial stage of the analysis consisted of preparing the databases separately: an individual database about health professionals (individual characteristics and readiness) and a database for each oral health team (work process). Descriptive analyses were performed to identify missing data ​​and questionnaires without identification, which were excluded. Absolute and relative frequencies were obtained for categorical variables, including all variables of the work process questionnaire, to assess the achievement of quality standards by the teams. The median and interquartile range (IQR, equal to the difference between the 75^th^ percentile and the 25^th^ percentile) were obtained for the total scores for organizational readiness for change and for the commitment and effectiveness constructs. Internal consistency was assessed by Cronbach’s alpha for the instrument as a whole and also for its dimensions. For the purposes of analysis of association, the organizational readiness constructs were transformed into categorical variables, taking the median of the scores as the cutoff point. Oral health teams with scores higher than the median were classified as having high effectiveness or commitment.

Following this, the set of work process variables was analyzed in order to identify groups of teams with similar work processes. To this end, a Latent Class Analysis was performed, given that all variables in the work process questionnaire were binary. This model assumed the existence of an underlying and unobserved categorical variable, which divided the population into exclusive and complete latent classes, inferred from a set of characteristics (17). We excluded questions that presented a low frequency of yes or no answers (less than 8.0%), as they were considered to be rare variables or with very unbalanced distribution and that did not contribute to differentiation between classes, but rather generated artificial or unstable classes.

During model adjustment, variables that did not contribute to class classification were excluded. Models with two and three latent classes were tested, and the final model was selected based on the lowest values ​​of the Akaike and Bayesian information criteria and on the highest entropy value (17). Marginal and conditional probabilities were analyzed to interpret the classes and characterize the teams according to their work process. Then, each team was assigned to a class according to the highest marginal probability observed, resulting in a new categorical variable that identified the class to which each team belonged. The classes were named according to the work process characteristics shared by the teams.

The work process database was linked to the database containing individual data using the unique variable identifying each team. Individual characteristics and levels of organizational readiness (effectiveness and commitment) were analyzed according to latent classes related to the work process. Pearson’s chi-square test was used to associate variables related to individual characteristics and organizational readiness for change with the work process of the teams. The Mann-Whitney U test was used to assess differences in length of service between teams according to the work process. A 95% significance level was adopted. Statistical analyses were performed using Stata version 18.

## Results

One hundred and fifty health professionals provided complete answers for all variables analyzed (response rate: 80.65%). The Organizational Readiness for Implementing Change instrument was answered by 163 (87.63%) of the 186 participants. Regarding the work process self-assessment instrument, 74 (86.05%) of the 86 registered teams responded. There was loss of data on the work process of one team due to incomplete completion and eight responses to the readiness questionnaire that did not contain team identification data.

Most professionals were female, aged between 30 and 50 years, with a predominance of dentists, professionals with permanent employment contracts and who had studied to postgraduate level (Table 1). Average length of service at the institution was 9.1 years (standard deviation: 9.5), while the median was 5.7 (IQR 11.8). The median of the total score of organizational readiness for change was 51 points (IQR 11). The median for effectiveness was 27 (IQR 6), while the median for commitment was 24 (IQR 5) (Table 1). The internal consistency of the readiness instrument, considering all items, was 0.9571, while it was 0.9174 for the effectiveness dimension and 0.9261 for the commitment dimension.

**Table 1:** Characteristics of professionals belonging to oral health teams participating in the MonitoraSB implementation survey and organizational readiness for change score. Minas Gerais, 2024 (n=163)

Variables	Total
	n (%)
Sex	
Female	138 (84.7%)
Male	25 (15.3%)
**Age** (**in years**)	
≤30	43 (26.4%)
>30 and ≤50	83 (50.9%)
>50	37 (22.7%)
Schooling	
High school education	31 (19.0%)
Technical education	40 (24.5%)
Graduation	35 (21.5%)
Postgraduate studies	57 (35.0%)
Professional category	
Dental surgeon	74 (45.4%)
Oral health auxiliary	58 (35.6%)
Oral health technician	31 (19.0%)
**Employment relationship**	
Permanent contract	101 (62.0%)
Temporary contract	62 (38.0%)
**Quantitative variables**	Median (IQR P25; P75)ª
Length of service (in years)	5.8 (11.8-1.5; 13.3)
Organizational readiness score	51 (11-44; 55)
Commitment score	24 (5-20; 25)
Effectiveness score	27 (6-24; 30)

ªIQR: interquartile range. P25: 25^th^ percentile. P75: 75^th^ percentile.

The quality standards of the work processes achieved by the oral health teams were presented (Table 2). In the intersectoral action/population participation subdimension, the teams had the lowest frequencies of meeting the standards. In the health service structure subdimension, the vast majority of the teams met the quality standards in relation to supplies and equipment, except for radiographic examination equipment. In the oral health team work process subdimension, the standards were largely achieved, being lower for the criteria that evaluated planning and use of visual resources, such as maps of the region and team meetings. Most teams met the standards for organizing access to services; however, measures to increase access, such as home visits and extending opening hours, were achieved by a smaller proportion of teams. In the oral health surveillance subdimension, just over half of the teams used data to characterize services or make situation diagnoses. The quality standards related to “promotion and prevention” actions were met by most teams. The approach to specific population groups, such as young children and their caregivers, as well as populations that use alcohol, tobacco and other drugs, occurred with a smaller proportion of teams. Most teams met the standards of the oral health diagnosis, treatment and rehabilitation subdimension.

**Table 2:** Distribution of oral health teams according to the achievement of the performance standard of work process practices in Primary Health Care. Minas Gerais, 2024 (n=74)

Work process practices	Quality standard achievement
	Yes n (%)
**Oral health management dimension**	
**Intersectoral action/population participation subdimension**	
The oral health team takes part regularly in intersectoral meetings with health professionals and other sectors.	48 (64.9%)
The oral health team takes part in social watchdog space meetings.	37 (50.0%)
The oral health team takes part in activities with the primary health center community to discuss local health problems, care offered and results obtained.	31 (41.9%)
Representatives of social movements and Brazilian National Health System users take part in Primary Health oral care team planning.	29 (39.2%)
Health service structure subdimension	
The oral health team has access to the internet via all computers used to record data on the Health Information System^a^.	70 (94.5%)
The oral health team has a computer for recording data on dental care^a^.	69 (93.3%)
The oral health team has complete equipment and instruments for its activities^a^.	66 (89.2%)
The primary health center has sufficient oral health supplies to carry out its actions in a regular manner^a^.	59 (79.7%)
The dental consulting rooms have adequate equipment for performing periapical/interproximal radiographs^a^.	25 (33.8%)
**Oral health team work process subdimension**	
The oral health team records dental care data on the Primary Care Health Information System^a^.	69 (93.3%)
The team uses the Primary Care Health Information System or management reports to plan and monitor its work^a^.	61 (82.4%)
The team uses indicators to monitor and plan oral health services.	59 (79.7%)
The actions of the oral health technician or auxiliary increase the population’s access to care and improve service efficiency and quality.	54 (72.9%)
The team dedicates part of its working hours to meetings with other Primary Care professionals^a^.	47 (63.5%)
The oral health team discusses cases and therapeutic projects.	47 (63.5%)
The team’s professionals meet monthly to evaluate results and actions planned.	37 (50.0%)
The Family Health Strategy keeps the “situation panel” up to date with data and indicators relating to its territory.	31 (41.9%)
**Oral health Primary Care provision dimension**	
**Oral health service access subdimension**	
The oral health team carries out service user welcoming^a^.	73 (98.6%)
Information on how the service works is made available in a clear and accessible manner^a^.	67 (90.5%)
There are criteria that differentiate urgent care from scheduled appointments^a^.	66 (89.1%)
There is special care for families in situations of social vulnerability.	56 (75.7%)
Home care is a regular activity of the oral health team.	47 (63.5%)
The primary care center offers extended hours for dental care.	24 (32.4%)
**Oral health surveillance subdimension**	
The oral health team uses data from the oral care form to describe the epidemiological profile and guide its actions.	50 (67.5%)
The team performs situation diagnosis taking into consideration population and epidemiological data and socioeconomic and cultural determinants to plan its actions.	41 (55.4%)
**Promotion and prevention subdimension**	
The oral health team provides oral hygiene guidance and supervised tooth brushing^a^.	70 (94.6%)
The oral health team carries out collective health education activities and group care^a^.	67 (90.5%)
Strategies to encourage healthy habits, respecting the local culture, are developed jointly with the Family Health Strategy^a^.	64 (86.5%)
The team undertakes systematic oral health education actions in schools and daycare centers^a^.	63 (85.1%)
There is oral health monitoring of children up to 5 years old.	49 (66.2%)
Educational groups are held with parents or those responsible for children’s oral health.	36 (48.6%)
The team, together with the Family Health Strategy, follows up on users of alcohol, tobacco and other drugs to reduce harm.	36 (48.6%)
**Oral health diagnosis, treatment and rehabilitation subdimension**	
The oral health team has a flowchart for referrals and counter-referrals in the Oral Health Care Network^a^.	70 (94.6%)
At least 60.0% of pregnant women registered receive dental care during prenatal care^a^.	69 (93.3%)
The team makes appointments on behalf of other Primary Care professionals to achieve joint care^a^.	69 (93.3%)
Examination of the oral cavity of elderly people is a routine practice in the service.	59 (79.7%)
The team monitors the oral health of adult women and men.	56 (75.7%)

^a^Questions excluded from the latent class analysis due to the high frequency of meeting the work process quality standard (above 92.0%) or because they do not contribute to class differentiation.

**Figure 2:**
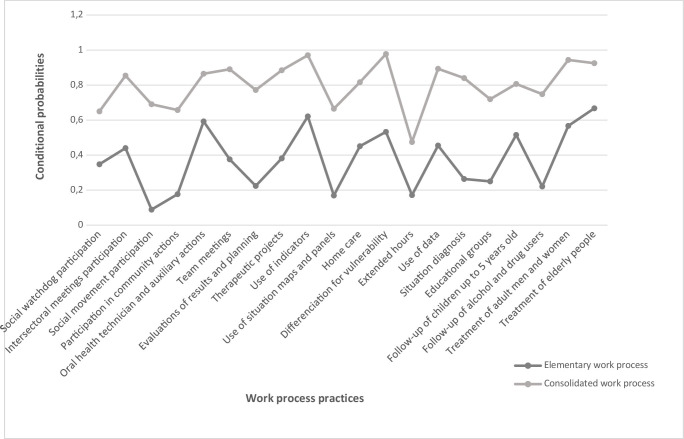
Conditional probabilities for the work process of oral health teams, according to identified latent classes. Minas Gerais, 2024

The latent class model adjustment included 20 variables related to the work process, which contributed to differentiation between classes. Seventeen variables were not included in the model (Table 2). The adjustment resulted in a two-class model, called “elementary work process” and “consolidated work process” (Figure 2). This model presented the best adjustment indicators according to the Akaike (1.71) and Bayesian (1.81) information criteria, when compared to the three-class model, in addition to the entropy value of 0.98. According to this model, 49.6% of the teams were classified as having an “elementary work process”, while 50.4% were classified as having a “consolidated work process”. Oral health teams tended to present a more positive work process, meeting quality standards when classified in the consolidated work process class (Figure 2).

The analysis of association between the variables revealed a higher percentage of teams with a consolidated work process among those with team members who were on temporary employment contracts (66.1%), compared to teams with professionals on permanent contracts (44.7%) (p-value 0.011). A higher percentage of oral health teams with a consolidated work process was observed among those in which the professionals were highly effective (60.8%) compared to teams with low effectiveness (44.7%) (p-value 0.049). Although not statistically significant (p-value 0.066), the higher percentage of teams with a consolidated work process (73.1%) stood out among those that had an oral health technician as a member of the team. There was no significant association between the other variables and the work process of the teams (Table 3).

**Table 3:** Frequency of oral health teams with an elementary or consolidated work process, according to individual characteristics and levels of effectiveness and commitment of oral health teams. Minas Gerais, 2024 (n=150)

Variables	Work process		
	Elementary	Consolidated	p-value
	n (%)	n (%)	
Sex			0.872
Male	11 (45.8)	13 (54.2)	
Female	60 (47.6)	66 (52.4)	
**Age** (**in years**)			0.309
≤30	15 (37.5)	25 (62.5)	
>30 and ≤50	37 (49.3)	38 (50.7)	
>50	19 (54.3)	16 (45.7)	
Schooling			0.402
High school education	18 (58.1)	13 (41.9)	
Technical education	15 (39.5)	23 (60.5)	
Graduation	12 (41.4)	17 (58.6)	
Postgraduate studies	26 (50.0)	26 (50.0)	
**Professional category**			0.066
Dental surgeon	35 (50.0)	35 (50.0)	
Oral health technician	7 (26.9)	19 (73.1)	
Oral health auxiliary	29 (53.7)	25 (46.3)	
**Length of service** (**in years**)			0.2975^a^
Median (interquartile range)	6.21(10.75)	4.46(13.48)	
**Employment relationship**			0.011
Permanent contract	52 (55.3)	42 (44.7)	
Temporary contract	19 (33.9)	37 (66.1)	
**Effectiveness construct**			0.049
Low	42 (55.3)	34 (44.7)	
High	29 (39.2)	45 (60.8)	
**Commitment construct**			0.149
Low	46 (52.3)	42 (47.7)	
High	25 (40.3)	37 (59.7)	

^a^Result of the Mann-Whitney U test.

## Discussion

The work process standards of the teams can be seen as health service outcomes, and can be modified with the incorporation of MonitoraSB through effective strategies. This study highlighted the work process as a central category for the analysis of outcomes of the implementation of MonitoraSB, combined with identification of facilitators and barriers. The teams presented a high level of organizational readiness, which demonstrated a favorable environment for the incorporation of MonitoraSB, with commitment, trust and the ability to incorporate innovation.

A limitation of this study was the use of a convenience sample. This criterion may have favored the selection of teams that were more willing to participate and interested in the topic, which may have influenced their responses, not necessarily reflecting the reality of oral health services in the municipalities. This limitation is relevant, since the contexts and individuals involved interact with each other and with the innovation itself, and may influence the process of implementing MonitoraSB (3,4,10).

The work process of teams can be modified with the incorporation of MonitoraSB, which enables qualification of practices through the analysis of indicators and the planning of actions. In turn, analysis of the work process can also demonstrate facilitators and barriers to MonitoraSB implementation. 

Access to computers and the internet was widespread, similar to that observed for Brazil as a whole (18). The use of information technology in health service provision, driven by digital transformation programs in the Brazilian National Health System (19, as well as the recording of data on Health Information Systems, favored by the use of electronic medical records, (20) are facilitators for the implementation of MonitoraSB. The use of management reports and indicators for the planning and monitoring of services strengthens the adoption of the monitoring proposal supported by digital tools (3,4).

Regular meetings for planning, monitoring, and evaluation were reported by the lowest proportion of teams. These meetings are essential spaces for structuring the service collaboratively, and their absence or lack of regularity may represent a barrier to the adoption of monitoring actions. On the other hand, monitoring can lead to them becoming a practice (20,21). Meeting the quality standards of the oral health surveillance work process was reported by the lowest proportion of teams when compared to the recording and use of health data. This finding converges with previous studies (8,22,23), which indicated that many teams associate these actions only with epidemiological surveys carried out among schoolchildren (22). The lack of routine use of indicators for planning and decision-making may indicate an aspect of the work process that can be improved through the incorporation of MonitoraSB (4).

The Health Services Structure dimension revealed the availability of adequate equipment and supplies for dental care, with the exception of radiographic equipment noted previously (23). Recent investments (24) have improved health center infrastructure, which has contributed to oral health promotion and care (8).

This study revealed a high level of organizational readiness for the incorporation of MonitoraSB, which may be associated with the aforementioned selection bias. Partnerships with higher education institutions can contribute to changes in the work process (22), in addition to favoring the organizational readiness of professionals, reducing internal barriers (25). Adequate communication and trust in change processes favor successful implementation (13). MonitoraSB’s collaborative implementation model (3) may have mobilized professionals, positively influencing organizational readiness.

Lack of knowledge and training regarding available resources constitute barriers to implementing changes, which results in lower organizational readiness among professionals (10). The Brazilian version of the Organizational Readiness for Implementing Change questionnaire proved to be an easy-to-apply instrument, with a high response rate and reliability similar to its validation study (16). This instrument appears to be effective in assessing the organizational readiness for change of health teams in the daily routine of public services for the implementation of new work processes, technologies and innovations in health (16), as is the case of MonitoraSB.

The highest percentage of teams with a consolidated work process was found among those comprised of professionals on temporary employment contracts, contradicting the initial hypothesis of the study. This type of contract, a growing trend in hiring staff in public health services (26), can generate turnover, overload professionals, weaken linkage and compromise the quality of care (27). In this study, it was observed that these professionals can demonstrate greater commitment to changes, possibly in order to maintain their employment relationship (28,29), favoring readiness. However, their turnover also impacts continuing education, interfering in the work process and in the performance of health actions (11), which can constitute a barrier to the incorporation of MonitoraSB.

Length of service and academic background were not statistically associated with team work process, contradicting the initial hypothesis. These findings seem to indicate that working conditions, employment relationships, and other contextual factors impact the organization of work, regardless of experience or professional qualifications (10). A higher proportion of oral health technicians was found in teams with a consolidated work process. Teams with these professionals demonstrated better work process organization and performance in health services (10). Expanding these teams represents the strategy for expanding and qualifying oral health care, in addition to favoring changes in work processes (30). This includes oral health monitoring and surveillance actions (5), which favors the incorporation of MonitoraSB.

In this study, teams with more organized work showed greater organizational readiness for change, evidenced by greater trust and cooperation among professionals regarding implementation of new practices, such as planning, monitoring, and evaluation. Reflection on professional practice, carried out in team meetings and strengthened by continuing health education actions, based on the routine use of health indicators, is a strategy for qualifying and driving changes in the oral health care model (20). In this scenario, an educational action for teams about MonitoraSB was adopted to promote its implementation (3).

Analysis of work process and organizational readiness for change of the teams proved to be fundamental in defining the MonitoraSB implementation strategies, being essential in all phases of the research, given its relevance and dynamic nature. In the initial phase, the teams with greater organizational readiness for change and less stable employment relationships presented more consolidated work processes more frequently. These results provided a basis for both the evaluation of the next stages of MonitoraSB implementation and for its adoption in other contexts, expanding its potential for application at a national level.

## References

[1] Agrasuta V, Thumbuntu T, Karawekpanyawong R, Panichkriangkrai W, Viriyathorn S, Reeponmaha T (2021). Progressive realisation of universal access to oral health services: What evidence is needed?. BMJ Glob Health.

[2] Ferreira RC, Chalub LLFH, Amaral JHL, Pinto RS, Santos JS, Campos FL (2025). Indicators for monitoring oral health services in primary care: content validation and measurability. Cien Saude Colet.

[3] Senna MIB, Ferreira RC (2024). MonitoraSB: uma proposta para o monitoramento e avaliação da saúde bucal na Atenção Primária à Saúde.

[4] Ferreira RC, Houri LCLF, Amaral JHL, Santos ME, Pinheiro EL, Gomes PM (2024). MonitoraSB: An innovation for monitoring and strengthening oral health in primary health care in Brazil. Rev Bras Epidemiol.

[5] Brasil (2004). Diretrizes da Política Nacional de Saúde Bucal.

[6] Brasil (2017). Portaria nº 2.436, de 21 de setembro de 2017. Aprova a Política Nacional de Atenção Básica, estabelecendo a revisão de diretrizes para a organização da Atenção Básica, no âmbito do Sistema Único de Saúde.

[7] Eshriqui I, Cordeiro L, Almeida LY, Sousa AAF, Paiva  FT, Vesga-Varela AL (2023). Utilizando ciência da implementação para avaliar intervenção em saúde mental: proposta metodológica. Acta Paul Enferm.

[8] Amorim LP, Senna MIB, Paula JS, Rodrigues LG, Chiari APG, Ferreira RC (2014). Oral health work process: disparity between teams in Brazil, 2014. Epidemiol Serv Saude.

[9] Damschroder LJ, Reardon CM, Widerquist MAO, Lowery J (2022). Conceptualizing outcomes for use with the Consolidated Framework for Implementation Research (CFIR): the CFIR Outcomes Addendum. Implement Sci.

[10] Damschroder LJ, Aron DC, Keith RE, Kirsh SR, Alexander JA, Lowery JC (2009). Fostering implementation of health services research findings into practice: A consolidated framework for advancing implementation science. Implement Sci.

[11] Weiner BJ (2009). A theory of organizational readiness for change. Implement Sci.

[12] Shea CM, Jacobs SR, Esserman DA, Bruce K, Weiner BJ (2014). Organizational readiness for implementing change: a psychometric assessment of a new measure. Implement Sci.

[13] Cunha-Cruz J, Milgrom P, Huebner CE, Scott J, Ludwig S, Dysert J (2017). Care delivery and compensation system changes: A case study of organizational readiness within a large dental care practice organization in the United States. BMC Oral Health.

[14] Hughes AM, Lin E, Hussain RA, Gibson G, Jurasic MM, Sharp LK (2023). The feasibility of academic detailing for acute oral pain management in outpatient dentistry: A pilot study. J Am Pharm Assoc.

[15] Brasil (2009). Avaliação para melhoria da qualidade da estratégia saúde da família.

[16] Bomfim RA, Braff EC, Frazão P (2020). Cross-cultural adaptation and psychometric properties of the Brazilian-Portuguese version of the organizational readiness for implementing change questionnaire. Rev Bras Epidemiol.

[17] Collins LM, Lanza ST (2010). Latent class and latent transition analysis: with applications in the social, behavioral, and health sciences.

[18] Comitê Gestor da Internet no Brasil (2023). TIC Saúde: Pesquisa sobre o uso das tecnologias de informação e comunicação nos estabelecimentos de saúde brasileiros – 2023 [Internet].

[19] Brasil (2024). Portaria GM/MS n° 3.232, de 1° de março de 2024. Altera a Portaria de Consolidação GM/MS nº 5, de 28 de setembro de 2017, para instituir o Programa SUS Digital.

[20] Bittar P (2023). UPAs, policlínicas e centros de especialidades poderão utilizar a nova versão do Prontuário Eletrônico do Cidadão [Internet].

[21] Silva ET, Ferreira RC, Diniz FC, Gomes MR, Martins AMEBL, Chalub LLFH (2024). Disparidades do protagonismo das equipes de saúde bucal no processo de trabalho na APS. Rev Saude Publica.

[22] Scherer CI, Scherer MDA, Chaves SCL, Menezes ELC (2018). O trabalho em saúde bucal na estratégia de saúde da família: uma difícil integração?. Saúde Debate.

[23] Gonçalves AJG, Pereira PHS, Monteiro V, Silva  MF, Baldani MH (2020). Estrutura dos serviços de saúde bucal ofertados na Atenção Básica no Brasil: diferenças regionais. Saúde Debate.

[24] Chaves SCL, Almeida AMFL, Rossi TRA, Santana SF, Barros SG, Santos CML (2003). Oral health policy in Brazil between 2003 and 2014: scenarios, proposals, actions, and outcomes. Cien Saude Colet.

[25] Mehta SN, Shenvi EC, Blair SL, Caudle A, Lowenstein LM, Kelly KJ (2023). Leveraging the multidisciplinary tumor board for dissemination of evidence-based recommendations on the staging and treatment of gastric cancer: a pilot study. Ann Surg Oncol.

[26] Morosini MVGC (2016). Precarização do trabalho: particularidades no setor saúde brasileiro. Trab Educ Saúde.

[27] Pereira AAC, Cunha CLF, Alvarenga EC, Lemos M, Bastos MSCBO, Silva KL (2023). Precarização do trabalho de enfermeiras: uma análise na atenção primária à saúde brasileira. Trab Educ Saúde.

[28] Vilela EN, Mafra LAS (2015). Estratégia saúde da família: contratação temporária e precarização nas relações de trabalho. Cad Est Interd.

[29] Damascena DM, Vale PRLF (2020). Tipologias da precarização do trabalho na atenção básica: um estudo netnográfico. Trab Educ Saúde.

[30] Brasil (2023). Portaria GM/MS n° 1.924, de 17 de novembro de 2023. Altera a Portaria de Consolidação GM/MS nº 6, de 28 de setembro de 2017.

