# Supplementary material for: Organizational readiness for change and work process of oral health teams in Primary Health Care: an exploratory study in the initial implementation phase of MonitoraSB, Minas Gerais, Brazil, 2024
Source: Epidemiol Serv Saude. 2025 Oct 20;34:e20240802. doi: 10.1590/S2237-96222025v34e20240802.en (PMC12536963; doi:10.1590/S2237-96222025v34e20240802.en)
Supplement: Translation

# Translation: Prontidão organizacional para mudança e processo de trabalho de equipes de saúde bucal na Atenção Primária à Saúde: estudo exploratório na fase inicial de implementação do MonitoraSB, Minas Gerais, 2024

**DOI:** 10.1590/S2237-96222025v34e20240802.pt

**Authors:** Maria Edileusa Santos, Raquel Conceição Ferreira, Elisa Lopes Pinheiro, Samira Suelen Andrade Vieira, Maria Inês Barreiros Senna

**Affiliations:** 1 Universidade Federal de Minas Gerais, Belo Horizonte, MG, Brasil; 2 Universidade Federal de Minas Gerais, Departamento de Odontologia Social e Preventiva, Belo Horizonte, MG, Brasil; 3 Universidade Federal de Minas Gerais, Departamento de Clínica, Patologia e Cirurgia Odontológicas, Belo Horizonte, MG, Brasil

**Keywords:** Pesquisa sobre Serviços de Saúde, Serviços de Saúde Bucal, Equipe de Saúde Bucal, Avaliação de Processos em Cuidados de Saúde, Estudo de Avaliação

## Abstract

**Objetivo:**

Investigar a associação das características individuais e da prontidão organizacional para mudança com o processo de trabalho das equipes de saúde bucal.

**Métodos:**

Tratou-se de estudo exploratório realizado na fase de alinhamento da implementação do MonitoraSB, proposta de monitoramento dos serviços de saúde bucal na Atenção Primária à Saúde, conduzida em 13 municípios de Minas Gerais, que abrangeu 86 equipes de saúde bucal. Avaliou-se o processo de trabalho dessas equipes como desfecho referente aos serviços de saúde por meio de questionário de autoavaliação. Determinantes da implementação (características individuais e prontidão organizacional para mudança) foram avaliados baseados no Consolidated Framework for Implementation Research. Frequências relativas foram obtidas para variáveis categóricas e mediana e distância interquartílica para os escores da prontidão. Análise de classes latentes agrupou as equipes segundo o processo de trabalho. Associações foram investigadas pelo teste qui-quadrado.

**Resultados:**

Participaram 74 equipes com 163 profissionais. Classificaram-se as equipes em “processo de trabalho elementar” (49,6%) e “consolidado” (50,4%) e apresentaram altos escores de prontidão organizacional (mediana: 51; distância interquartílica: 11). Observou-se o maior percentual de equipes com processo de trabalho consolidado naquelas cujos profissionais eram contratados (66,1%), em comparação às equipes com efetivos (44,7%) (p-valor 0,011), e naquelas cujos profissionais apresentavam alta eficácia (60,8%) comparado àquelas com baixa eficácia (44,7%) (p-valor 0,049).

**Conclusão:**

Equipes com maior prontidão organizacional e vínculos de trabalho menos estáveis apresentaram, com maior frequência, processos de trabalho mais consolidados. A prontidão organizacional deve ser considerada para favorecer a implementação de iniciativas de monitoramento, como o MonitoraSB.

**Aspectos éticos**

A presente pesquisa respeitou os princípios éticos, obtendo os seguintes dados de aprovação:

Comitê de ética em pesquisa: Universidade Federal de Minas Gerais

Número do parecer: 5.326.307

Data de aprovação: 1/4/2022

Certificado de apresentação de apreciação ética: 55573922.3.0000.5149

Registro do consentimento livre e esclarecido: Todos os participantes o assinaram.

## Introdução

A oferta e o financiamento da saúde bucal são globalmente afetados pela falta de indicadores para o monitoramento e a avaliação dos serviços (1). No Brasil, esses indicadores são limitados e impactados por mudanças nas iniciativas de avaliação da Atenção Primária à Saúde ao longo do tempo (2). O MonitoraSB surge como uma inovação para o monitoramento e a avaliação dos serviços de saúde bucal na Atenção Primária à Saúde que busca suprir essa lacuna (2). É composto pela matriz de 54 indicadores elaborada a partir de dados disponibilizados pelo Sistema de Informação em Saúde para a Atenção Básica e ferramentas digitais: o painel interativo e a calculadora (3,4).

O monitoramento e a avaliação são diretrizes da Política Nacional de Saúde Bucal para aprimorar os serviços do Sistema Único de Saúde (5). Equipes de saúde bucal devem gerar, alimentar e garantir a qualidade dos registros nos sistemas de informação em saúde. Além disso, devem participar de reuniões para acompanhar e discutir o planejamento e a avaliação das ações da equipe, utilizando os dados disponíveis para ajustar seu processo de trabalho (6).

A implementação do MonitoraSB propõe a participação dos profissionais como corresponsáveis e sujeitos ativos do processo de monitoramento (3). A equipe analisa e interpreta as informações fornecidas pelos indicadores do MonitoraSB e participa da discussão e do planejamento de ações para enfrentar as dificuldades reveladas pelo monitoramento, tendo como meta a melhoria do cuidado ofertado (3). Essa escolha assume que o trabalho em saúde se caracteriza por um processo coletivo, colaborativo e institucional (5), orientado pelas tomadas de decisões informadas sobre as necessidades da população e o desempenho dos serviços (7).

Na pesquisa de implementação, os desfechos dos serviços de saúde podem revelar o efeito da adoção de novas práticas ou intervenções (2). Dessa forma, o processo de trabalho das equipes de saúde bucal pode ser desfecho sensível às intervenções voltadas ao monitoramento de indicadores (8). O processo de trabalho das equipes pode variar conforme características dos profissionais, dos serviços e das demandas (5,8).

Identificar determinantes da implementação visa compreender as barreiras e os facilitadores para a implementação efetiva da inovação (9). O Consolidated Framework for Implementation Research é uma das estruturas mais utilizadas para isso (9). Esta apresenta 37 construtos organizados em cinco domínios: características da intervenção, contexto externo, contexto interno, características dos indivíduos envolvidos e processo de implementação (10).

O contexto interno abrange características estruturais, rede de relações, comunicação, cultura, clima e prontidão organizacional, que podem interagir e impactar o sucesso da implementação (10). A prontidão organizacional para mudança é um construto multinível que reflete o estado psicológico e comportamental compartilhado por uma equipe e está relacionado ao comprometimento e à capacidade de implementar mudanças (11). Além de ser fator preditivo para o sucesso da implementação, essa prontidão é dinâmica e pode ser modificada durante o processo (10-12). Apesar da sua relevância, é pouco estudada entre profissionais de saúde bucal (11,13,14). Características individuais, como conhecimento e convicções sobre a intervenção, identificação com a organização e outros atributos pessoais, também afetam a implementação (10). Os determinantes em uma implementação podem ser transitórios e sujeitos a mudanças, o que ressalta a importância de seu monitoramento (10).

Caracterizar os determinantes individuais e contextuais da implementação e o processo de trabalho das equipes é essencial na fase inicial de implementação do MonitoraSB (2). Essa análise pode orientar a elaboração de estratégias eficazes para sua implementação bem-sucedida, promovendo mudanças no processo de trabalho (2).

O objetivo deste estudo foi descrever o processo de trabalho das equipes de saúde bucal na fase inicial da implementação do MonitoraSB e caracterizar esses profissionais quanto ao perfil demográfico, à atuação profissional e à prontidão organizacional para mudança. Além disso, investigou-se a associação das características individuais e da prontidão organizacional para mudança com o processo de trabalho das equipes. A hipótese testada foi que um maior percentual de equipes com processos de trabalho mais consolidados seria observado quando seus profissionais apresentassem maior qualificação, vínculos estáveis e maior prontidão organizacional para a mudança.

## Métodos

### Delineamento e amostra do estudo

Tratou-se de estudo exploratório baseado em dados da fase inicial da pesquisa de implementação do MonitoraSB, aprovado pelo Comitê de Ética em Pesquisa da Universidade Federal de Minas Gerais. Utilizaram-se métodos mistos e está em curso em 13 municípios de Minas Gerais (12 municípios do interior e a capital). A seleção dos municípios foi realizada por conveniência, considerando parcerias com a Faculdade de Odontologia da Universidade Federal de Minas Gerais e municípios de origem de egressos do mestrado profissional em odontologia em saúde pública. Os municípios foram agrupados pelo porte populacional, segundo os dados de 2022 do Instituto Brasileiro de Geografia e Estatística, e classificados em grupos Minas (até 40 mil habitantes) e Gerais (população com mais de 100 mil habitantes) (Figura 1).

**Figura 1:** Municípios participantes da pesquisa de implementação do MonitoraSB dos grupos Minas e Gerais. Minas Gerais, 2022

A amostra compreendeu 86 equipes de saúde bucal indicadas pelos gestores municipais e totalizou 186 profissionais, incluindo cirurgiões-dentistas, técnicos e auxiliares em saúde bucal.

### Variáveis e fonte de dados 

O processo de trabalho das equipes de saúde bucal foi o desfecho referente aos serviços de saúde, analisado por um instrumento de autoavaliação baseado nas diretrizes da Política Nacional de Saúde Bucal, da Política Nacional de Atenção Básica, do Caderno de Autoavaliação para Melhoria do Acesso e da Qualidade da Atenção Básica e do Programa de Melhoria do Acesso e Qualidade da Atenção Básica (2). Foi validado por 19 especialistas, por meio da técnica Delphi modificada, com índice de validade de conteúdo >75% (3). O instrumento utilizou padrões de qualidade predefinidos para avaliar o desempenho das equipes (15) e era composto por 37 afirmativas organizadas em duas dimensões e sete subdimensões, conforme o modelo de avaliação da efetividade da atenção em saúde bucal adotado pelo MonitoraSB (3). Cada padrão foi acompanhado por uma afirmativa, e o profissional da equipe selecionou “sim” ou “não”, de acordo com o alcance do padrão. Esse instrumento foi respondido por um dos profissionais da equipe.

Os determinantes de implementação (características individuais e prontidão organizacional para mudança) foram avaliados por todos os profissionais das equipes. As características individuais analisadas foram: sexo, idade, categoria profissional (cirurgião-dentista, técnico, auxiliar em saúde bucal), escolaridade (ensino médio, técnico, graduação, pós-graduação), vínculo de trabalho (contrato temporário, efetivo) e tempo de serviço na instituição. A prontidão organizacional foi avaliada pela versão brasileira validada do Organizational Readiness for Implementing Change (3,16), composta por 11 afirmativas em escala de Likert de 5 pontos (“1 discordo totalmente” a “5 concordo totalmente”). O escore da prontidão organizacional para a mudança foi obtido pela soma da pontuação total (pontuação entre 11 e 55) e das questões referentes aos construtos compromisso (pontuação entre 5 e 25) e eficácia (pontuação entre 6 e 30). 

### Procedimentos de coleta de dados e minimização dos vieses

A coleta de dados aconteceu de março de 2023 a fevereiro de 2024. Os instrumentos foram enviados por e-mail individualmente para cada profissional e compartilhados via WhatsApp pelas referências locais, profissionais que coordenam a implementação do MonitoraSB nos municípios. Alertas foram enviados para aumentar a participação e a adesão dos envolvidos. Os questionários continham instruções detalhadas para o correto preenchimento. Durante a coleta, as referências locais e os pesquisadores estiveram disponíveis para o esclarecimento de dúvidas.

### Análise estatística

A etapa inicial da análise consistiu na preparação das bases de dados separadamente: base de dados individuais dos profissionais (características individuais e prontidão) e base de dados para cada equipe de saúde bucal (processo de trabalho). Análises descritivas foram realizadas para identificação de valores faltantes e questionários sem identificação, que foram excluídos. Foram obtidas frequências absoluta e relativa para as variáveis categóricas, incluindo todas as variáveis do questionário de processo de trabalho, para avaliar o alcance dos padrões de qualidade pelas equipes. A mediana e a distância interquartílica (DI, igual à diferença entre percentil 75 e percentil 25) foram obtidas para os escores totais de prontidão organizacional para mudança e para os construtos compromisso e eficácia. A consistência interna foi avaliada pelo alfa de Cronbach para o instrumento completo e suas dimensões. Para fins da análise de associação, os construtos da prontidão organizacional foram transformados em variáveis categóricas, assumindo como ponto de corte a mediana dos escores. Equipes de saúde bucal com escores maiores do que a mediana foram classificadas como eficácia ou compromisso altos. 

Em seguida, a análise do conjunto de variáveis de processo de trabalho foi realizada buscando identificar agrupamentos de equipes com processos de trabalho semelhantes. Para isso, foi realizada uma Análise de Classes Latentes, tendo em vista que todas as variáveis do questionário de processo de trabalho eram binárias. Esse modelo assumiu a existência de uma variável categórica subjacente e não observada, que dividiu a população em classes latentes exclusivas e completas, inferidas a partir de um conjunto de características (17). Foram excluídas questões que apresentaram baixa frequência de respostas não ou sim (menores de 8,0%), consideradas variáveis raras ou com distribuição muito desequilibrada e que não contribuíram para a discriminação entre classes, gerando classes artificiais ou instáveis. 

Durante o ajuste dos modelos, foram excluídas variáveis que não contribuíram para a classificação das classes. Modelos com duas e três classes latentes foram testados, sendo selecionado o modelo final com base nos menores valores dos critérios de informação de Akaike e bayesiano e no maior valor de entropia (17). Analisaram-se as probabilidades marginais e condicionais para interpretar as classes e caracterizar as equipes segundo seu processo de trabalho. Em seguida, cada equipe foi atribuída a uma classe de acordo com a maior probabilidade marginal observada, resultando em nova variável categórica que identificou a classe a qual cada equipe pertencia. As classes foram denominadas segundo as características de processo de trabalho compartilhadas pelas equipes. 

A base de dados de processo de trabalho foi vinculada à base contendo dados individuais pela variável única de identificação de cada equipe. As características individuais e os níveis de prontidão organizacional (eficácia e compromisso) foram analisados segundo as classes latentes referentes ao processo de trabalho. O teste qui-quadrado de Pearson foi utilizado para associar as variáveis referentes às características individuais e à prontidão organizacional para mudança e o processo de trabalho das equipes. O teste U de Mann-Whitney foi empregado para avaliar as diferenças do tempo de trabalho entre as equipes, segundo o processo de trabalho. Foi adotado o nível de significância de 95%. As análises estatísticas foram realizadas no software Stata, versão 18.

## Resultados

Cento e cinquenta profissionais apresentaram respostas completas para todas as variáveis analisadas (taxa de resposta: 80,65%). O instrumento Organizational Readiness for Implementing Change foi respondido por 163 (87,63%) dos 186 participantes. Para o instrumento de autoavaliação do processo de trabalho, das 86 equipes cadastradas, 74 (86,05%) responderam. Houve perda de dados sobre o processo de trabalho de uma equipe por preenchimento incompleto e de oito respostas do questionário de prontidão que não continham dados de identificação da equipe. 

A maioria dos profissionais era do sexo feminino, com idade entre 30-50 anos, com a predominância de cirurgiões-dentistas, profissionais efetivos e com pós-graduação (Tabela 1). O tempo médio de serviço na instituição foi 9,1 anos (desvio-padrão: 9,^5^), e a mediana, 5,7 (DI 11,^8^) A mediana do escore total da prontidão organizacional para mudança foi 51 pontos (DI 11). A mediana da eficácia foi 27 (DI 6), e do comprometimento, 24 (DI 5) (Tabela 1). A consistência interna do instrumento de prontidão, considerando todos os itens, foi 0,9571; das dimensões eficácia, 0,9174; e de comprometimento, 0,9261.

**Tabela 1:** Características dos profissionais das equipes de saúde bucal participantes da pesquisa de implementação do MonitoraSB e escore da prontidão organizacional para mudança. Minas Gerais, 2024 (n=163)

Variáveis	Total
	n (%)
Sexo	
Feminino	138 (84,7%)
Masculino	25 (15,3%)
**Idade** (**em anos**)	
≤30	43 (26,4%)
>30 e ≤50	83 (50,9%)
>50	37 (22,7%)
Escolaridade	
Ensino médio	31 (19,0%)
Ensino técnico	40 (24,5%)
Graduação	35 (21,5%)
Pós-graduação	57 (35,0%)
**Categoria profissional**	
Cirurgião-dentista	74 (45,4%)
Auxiliar em saúde bucal	58 (35,6%)
Técnico em saúde bucal	31 (19,0%)
**Vínculo de trabalho**	
Efetivo	101 (62,0%)
Contratado	62 (38,0%)
**Variáveis quantitativas**	Mediana (DI P25; P75)ª
Tempo de serviço (em anos)	5,8 (11,8-1,5; 13,3)
Escore prontidão organizacional	51 (11-44; 55)
Escore de comprometimento	24 (5-20; 25)
Escore de eficácia	27 (6-24; 30)

ªDI: distância interquartílica. P25: percentil 25. P75: percentil 75.

Apresentaram-se os padrões de qualidade dos processos de trabalho alcançados pelas equipes de saúde bucal (Tabela 2). Na subdimensão ação intersetorial/participação popular, as equipes tiveram as menores frequências de atendimento aos padrões. Na subdimensão estrutura dos serviços de saúde, a grande maioria das equipes atendeu aos padrões de qualidade em relação aos insumos e equipamentos, exceto para os equipamentos de exames radiográficos. Na subdimensão processo de trabalho das equipes de saúde bucal, os padrões foram amplamente alcançados, sendo menores para os critérios que avaliavam o planejamento e uso de recursos visuais, como mapas da região e reuniões de equipe. A maioria das equipes atendeu aos padrões de organização do acesso aos serviços; entretanto, medidas de ampliação do acesso, como visitas domiciliares e extensão do horário de funcionamento, foram alcançadas pela menor proporção das equipes. Na subdimensão vigilância em saúde bucal, pouco mais da metade das equipes utilizou dados para caracterização dos serviços ou diagnósticos situacionais. Os padrões de qualidade relativos às ações de “promoção e prevenção” foram atendidos pela maioria das equipes. A abordagem de grupos populacionais específicos, como crianças pequenas e seus responsáveis, bem como populações usuárias de álcool, tabaco e outras drogas, ocorreu com proporção menor das equipes. A maioria das equipes atendeu aos padrões da subdimensão diagnóstico, tratamento e reabilitação em saúde bucal.

**Tabela 2:** Distribuição das equipes de saúde bucal conforme o alcance do padrão de desempenho das práticas do processo de trabalho na Atenção Primária à Saúde. Minas Gerais, 2024 (n=74)

Práticas do processo de trabalho	Alcance do padrão de qualidade
	n (%)
**Dimensão gestão de saúde bucal**	
**Subdimensão ação intersetorial/participação popular**	
A equipe de saúde bucal participa regularmente de reuniões intersetoriais com profissionais da saúde e de outros setores.	48 (64,9%)
A equipe de saúde bucal participa de reuniões de instâncias de controle social.	37 (50,0%)
A equipe de saúde bucal participa de atividades com a comunidade da unidade básica de saúde para discutir problemas de saúde locais, a assistência oferecida e os resultados obtidos.	31 (41,9%)
Representantes de movimentos sociais e usuários do Sistema Único de Saúde participam no planejamento das equipes de saúde bucal na Atenção Primária à Saúde.	29 (39,2%)
**Subdimensão estrutura dos serviços de saúde**	
A equipe de saúde bucal tem acesso à internet em todos os computadores usados para registrar dados no Sistema de Informação em Saúde^a^.	70 (94,5%)
A equipe de saúde bucal tem computador para registrar os dados dos atendimentos odontológicos^a^.	69 (93,3%)
A equipe de saúde bucal possui equipamentos e instrumentais completos para suas atividades^a^.	66 (89,2%)
A unidade básica de saúde tem insumos de saúde bucal suficientes para realizar as ações regularmente^a^.	59 (79,7%)
Os consultórios odontológicos possuem equipamentos adequados para realizar radiografias periapicais/interproximais^a^.	25 (33,8%)
**Subdimensão processo de trabalho das equipes de saúde bucal**	
A equipe de saúde bucal registra os atendimentos odontológicos no Sistema de Informação em Saúde para a Atenção Básica^a^.	69 (93,3%)
A equipe utiliza o Sistema de Informação em Saúde para a Atenção Básica ou relatórios gerenciais para planejar e monitorar o trabalho^a^.	61 (82,4%)
A equipe utiliza indicadores para o monitoramento e planejamento dos serviços de saúde bucal.	59 (79,7%)
As ações do técnico ou auxiliar em saúde bucal aumentam o acesso da população aos cuidados e melhoram a eficiência e qualidade dos serviços.	54 (72,9%)
A equipe dedica parte da carga horária para reuniões com outros profissionais da Atenção Básica^a^.	47 (63,5%)
A equipe de saúde bucal discute casos e projetos terapêuticos.	47 (63,5%)
Os profissionais da equipe se reúnem mensalmente para avaliar resultados e ações planejadas.	37 (50,0%)
A estratégia de saúde da família mantém atualizado o “painel de situação” com dados e indicadores do território.	31 (41,9%)
**Dimensão provimento de Atenção Primária à saúde bucal**	
**Subdimensão acesso aos serviços de saúde bucal**	
A equipe de saúde bucal realiza o acolhimento dos usuários^a^.	73 (98.6%)
As informações sobre o funcionamento do serviço são disponibilizadas de forma clara e acessível^a^.	67 (90,5%)
Há critérios que diferenciam o pronto atendimento de atendimentos programados^a^.	66 (89,1%)
Existe atenção especial para famílias em situação de vulnerabilidade social.	56 (75,7%)
A atenção domiciliar é uma atividade regular da equipe de saúde bucal.	47 (63,5%)
A unidade básica de saúde oferece horários estendidos para atendimento odontológico.	24 (32,4%)
**Subdimensão vigilância em saúde bucal**	
A equipe de saúde bucal utiliza dados da ficha odontológica para traçar o perfil epidemiológico e orientar suas ações.	50 (67,5%)
A equipe realiza diagnóstico situacional considerando dados populacionais, epidemiológicos e determinantes socioeconômicos e culturais para planejar suas ações.	41 (55,4%)
**Subdimensão promoção e prevenção**	
A equipe de saúde bucal realiza orientação de higiene bucal e escovação dental supervisionada^a^.	70 (94,6%)
A equipe de saúde bucal realiza atividades coletivas de educação em saúde e atendimentos em grupo^a^.	67 (90,5%)
Estratégias para estimular hábitos saudáveis, respeitando a cultura local, são desenvolvidas em parceria com a estratégia de saúde da família^a^.	64 (86,5%)
A equipe promove ações sistemáticas de educação em saúde bucal em escolas e creches^a^.	63 (85,1%)
Há acompanhamento da saúde bucal de crianças de até 5 anos.	49 (66,2%)
São realizados grupos educativos com pais ou responsáveis pela saúde bucal infantil.	36 (48,6%)
A equipe, junto com a estratégia de saúde da família, acompanha usuários de álcool, tabaco e outras drogas para redução de danos.	36 (48,6%)
**Subdimensão diagnóstico, tratamento e reabilitação em saúde bucal**	
A equipe de saúde bucal possui fluxo de encaminhamento com referência e contrarreferência na Rede de Atenção à Saúde Bucal^a^.	70 (94,6%)
Pelo menos 60,0% das gestantes cadastradas recebem atendimento odontológico durante o pré-natal^a^.	69 (93,3%)
A equipe solicita agendamentos para outros profissionais de Atenção Básica para cuidado compartilhado^a^.	69 (93,3%)
O exame da cavidade bucal em idosos é uma prática rotineira no serviço.	59 (79,7%)
A equipe acompanha a saúde bucal de mulheres e homens adultos.	56 (75,7%)

^a^Questões excluídas da análise de classes latentes devido à alta frequência de atendimento ao padrão de qualidade do processo de trabalho (acima de 92,0%) ou por não contribuírem para a discriminação das classes.

O ajuste do modelo de classes latentes incluiu 20 variáveis referentes ao processo de trabalho, as quais contribuíram para a discriminação entre as classes. Dezessete variáveis não compuseram o modelo (Tabela 2). O ajuste resultou em um modelo de duas classes, denominadas “processo de trabalho elementar” e “processo de trabalho consolidado” (Figura 2). Esse modelo apresentou os melhores indicadores de ajuste segundo os critérios de informação de Akaike (1,71) e bayesiano (1,81), quando comparado ao modelo de três classes, além do valor de entropia de 0,98. De acordo com esse modelo, as proporções de equipes classificadas como “processo de trabalho elementar” e “processo de trabalho consolidado” foram 49,6% e 50,4%. As equipes de saúde bucal tendem a apresentar processo de trabalho mais positivo, com alcance dos padrões de qualidade quando classificadas na classe processo de trabalho consolidado (Figura 2).

**Figura 2:** Probabilidades condicionadas para o processo de trabalho das equipes de saúde bucal, segundo classes latentes identificadas. Minas Gerais, 2024

A análise de associação entre as variáveis revelou maior percentual de equipes com processo de trabalho consolidado entre aquelas cujos profissionais eram contratados (66,1%), em comparação às equipes com profissionais efetivos (44,7%) (p-valor 0,011). Observou-se maior percentual de equipes de saúde bucal com processo de trabalho consolidado entre aquelas nas quais os profissionais apresentavam alta eficácia (60,8%) comparado às equipes com baixa eficácia (44,7%) (p-valor 0,049). Embora não estatisticamente significativo (p-valor 0,066), destacou-se o maior percentual de equipes com processo de trabalho consolidado (73,1%) naquelas que possuíam o técnico de saúde bucal como membro da equipe. Não houve associação significativa entre as demais variáveis e o processo de trabalho das equipes (Tabela 3).

**Tabela 3:** Frequência de equipes de saúde bucal com processo de trabalho elementar ou consolidado, segundo características individuais e níveis de eficácia e compromisso das equipes de saúde bucal. Minas Gerais, 2024 (n=150)

Variáveis	Processo de trabalho		
	Elementar	Consolidado	
	n (%)	n (%)	p-valor
Sexo			0,872
Masculino	11 (45,8)	13 (54,2)	
Feminino	60 (47,6)	66 (52,4)	
**Idade** (**em anos**)			0,309
≤30	15 (37,5)	25 (62,5)	
>30 e ≤50	37 (49,3)	38 (50,7)	
>50	19 (54,3)	16 (45,7)	
Escolaridade			0,402
Ensino médio	18 (58,1)	13 (41,9)	
Ensino técnico	15 (39,5)	23 (60,5)	
Graduação	12 (41,4)	17 (58,6)	
Pós-graduação	26 (50,0)	26 (50,0)	
**Categoria profissional**			0,066
Cirurgião-dentista	35 (50,0)	35 (50,0)	
Técnico em saúde bucal	7 (26,9)	19 (73,1)	
Auxiliar em saúde bucal	29 (53,7)	25 (46,3)	
**Tempo de serviço** (**em anos**)			0,2975^a^
Mediana (distância interquartílica)	6,21(10,75)	4,46(13,48)	
**Vínculo de trabalho**			0,011
Efetivo	52 (55,3)	42 (44,7)	
Contratado	19 (33,9)	37 (66,1)	
**Construto eficácia**			0,049
Baixa	42 (55,3)	34 (44,7)	
Alta	29 (39,2)	45 (60,8)	
**Construto comprometimento**			0,149
Baixo	46 (52,3)	42 (47,7)	
Alto	25 (40,3)	37 (59,7)	

^a^Resultado do teste U de Mann-Whitney.

## Discussão

Os padrões do processo de trabalho das equipes se configuram como desfechos dos serviços de saúde, podendo ser modificados com a incorporação do MonitoraSB por meio de estratégias eficazes. Este estudo destacou o processo de trabalho como categoria central para a análise de desfechos da implementação do MonitoraSB, aliada com a identificação de facilitadores e barreiras. As equipes apresentaram alto nível de prontidão organizacional o que evidenciou ambiente favorável à incorporação do MonitoraSB, com comprometimento, confiança e capacidade para incorporar a inovação. 

Uma limitação deste estudo foi o uso de amostra de conveniência. Esse critério pode ter favorecido a seleção de equipes mais dispostas a participar, com interesse no tema, o que pode ter influenciado suas respostas, não refletindo, necessariamente, a realidade dos serviços de saúde bucal nos municípios. Essa limitação é relevante, pois os contextos e indivíduos envolvidos interagem entre si e com a inovação podendo influenciar o processo de implementação do MonitoraSB (3,4,10).

O processo de trabalho das equipes pode ser modificado com a incorporação do MonitoraSB, o que possibilita a qualificação das práticas por meio da análise de indicadores e do planejamento das ações. Por sua vez, a análise do processo de trabalho também pode demonstrar facilitadores e barreiras à implementação do MonitoraSB. 

O acesso a computadores e internet é amplo, semelhante ao observado no país (18). A informatização da saúde impulsionada por programas de transformação digital no Sistema Único de Saúde (19) e as práticas de registro de dados em Sistemas de Informação em Saúde, favorecida com o uso dos prontuários eletrônicos, (20) são facilitadores para implementação do MonitoraSB. O uso de relatórios gerenciais e indicadores para o planejamento e monitoramento dos serviços fortalece a adoção da proposta de monitoramento apoiada em ferramentas digitais (3,4).

Reuniões regulares para planejamento, monitoramento e avaliação foram relatadas pela menor proporção de equipes. Essas reuniões são espaços essenciais para estruturar o serviço de forma colaborativa, e sua ausência ou pouca regularidade pode representar uma barreira à adoção de ações de monitoramento. Por sua vez, pode se tornar prática a partir do monitoramento (20,21). Os padrões de qualidade do processo de trabalho na vigilância em saúde bucal foram relatados pela menor proporção de equipes quando comparada ao registro e uso de dados de saúde. Este achado converge com estudos anteriores (8,22,23), os quais apontaram que muitas equipes associam essas ações apenas aos levantamentos epidemiológicos realizados em escolares (22). A falta de uso rotineiro de indicadores para o planejamento e a tomada de decisões pode indicar aspecto do processo de trabalho que pode ser aprimorado a partir da incorporação do MonitoraSB (4).

A dimensão Estrutura dos serviços de saúde revelou a disponibilidade de equipamentos e insumos adequados para a assistência odontológica, com exceção para os equipamentos radiográficos observada anteriormente (23). Investimentos recentes (24) melhoraram a infraestrutura nas unidades de saúde, o que contribuiu para a promoção e assistência à saúde bucal (8).

Este estudo revelou o alto nível de prontidão organizacional para a incorporação do MonitoraSB, o que pode estar associado ao viés de seleção já mencionado. Parcerias com instituições de ensino superior podem contribuir para mudanças no processo de trabalho (22), além de favorecer a prontidão organizacional dos profissionais, reduzindo barreiras internas (25). A comunicação adequada e a confiança nos processos de mudança favorecem a implementação bem-sucedida (13). O modelo colaborativo de implementação do MonitoraSB (3) pode ter mobilizado os profissionais, influenciando positivamente a prontidão organizacional. 

A falta de conhecimentos e de capacitação sobre os recursos disponíveis constituem-se em barreiras para a implementação de mudanças, o que resulta em menor prontidão organizacional dos profissionais (10). A versão brasileira do Organizational Readiness for Implementing Change demonstrou ser instrumento de fácil aplicação, com alta taxa de resposta e confiabilidade semelhante ao estudo de validação (16). Esse instrumento parece ser eficaz para avaliação da prontidão organizacional para mudança das equipes de saúde no cotidiano dos serviços públicos para implementação de novos processos de trabalho, tecnologias e inovações em saúde (16), como é o caso do MonitoraSB.

O maior percentual de equipes com processo de trabalho consolidado foi observado entre aquelas cujos profissionais eram contratados, contrariando a hipótese inicial do estudo. Esse tipo de vínculo, tendência crescente nas contratações em serviços públicos de saúde (26), pode gerar rotatividade, sobrecarregar os profissionais, fragilizar vínculos e comprometer a qualidade da assistência (27). Neste estudo, observou-se que esses profissionais podem demonstrar maior comprometimento com as mudanças, possivelmente para manter o vínculo empregatício (28,29), favorecendo a prontidão. Porém, a rotatividade daqueles também impacta a educação permanente interferindo no processo de trabalho e no desempenho das ações de saúde (11), o que pode constituir barreira para a incorporação do MonitoraSB. 

O tempo de trabalho e a formação acadêmica não foram estatisticamente associados ao processo de trabalho das equipes, contradizendo a hipótese inicial. Esses achados parecem indicar que condições de trabalho, vínculos profissionais e outros fatores contextuais impactam a organização do trabalho, independentemente da experiência ou da qualificação profissional (10). Nas equipes com processo de trabalho consolidado, observou-se maior proporção de técnicos de saúde bucal. As equipes com esses profissionais demonstraram melhor organização do processo de trabalho e desempenho nos serviços de saúde (10). A expansão dessas equipes representa a estratégia para ampliar e qualificar a atenção à saúde bucal, além de favorecer as mudanças nos processos de trabalho (30). Isso inclui as ações de monitoramento e vigilância em saúde bucal (5), o que favorece a incorporação do MonitoraSB.

Neste estudo, equipes com trabalho mais organizado apresentaram maior prontidão organizacional para mudança, evidenciada pela maior confiança e cooperação entre os profissionais, para a implementação de novas práticas, como planejamento, monitoramento e avaliação. A reflexão sobre a prática profissional, realizada em reuniões de equipe e fortalecida por ações de educação permanente em saúde, alicerçada no uso rotineiro de indicadores de saúde, é uma estratégia para qualificar e impulsionar mudanças no modelo de atenção à saúde bucal (20). Nesse cenário, uma ação educativa sobre o MonitoraSB para as equipes foi adotada para promover a sua implementação (3).

A análise do processo de trabalho e da prontidão organizacional para mudança das equipes revelou-se fundamental na definição das estratégias de implementação do MonitoraSB, sendo essencial em todas as fases da pesquisa, dada a sua relevância e natureza dinâmica. Na fase inicial, as equipes com maior prontidão organizacional para mudança e vínculos de trabalho menos estáveis apresentaram, com mais frequência, processos de trabalho mais consolidados. Esses resultados forneceram base tanto para a avaliação das próximas etapas da implementação do MonitoraSB quanto para sua adoção em outros contextos, ampliando seu potencial de aplicação em nível nacional.
